# Innovation in Healthy and Sustainable Food Product Development for Health and Aged Care: A Scoping Review

**DOI:** 10.3390/foods11223604

**Published:** 2022-11-11

**Authors:** Judi Porter, Nathan Cook, Ranil Coorey, Don Gunasekera, Martin Hensher, Deborah A. Kerr, Christina M. Pollard, Serene Yoong, Gary Dykes, Mark Lawrence

**Affiliations:** 1 Institute for Physical Activity and Nutrition (IPAN), School of Exercise and Nutrition Sciences, Deakin University, Geelong, VIC 3220, Australia; 2 Faculty of Science and Engineering, School of Molecular and Life Sciences, Curtin University, Perth, WA 6102, Australia; 3 Faculty of Science, Engineering and Built Environment, Centre for Regional and Rural Futures, Deakin University, Geelong, VIC 3220, Australia; 4 Menzies Institute for Medical Research, University of Tasmania, Hobart, TAS 7000, Australia; 5 Faculty of Health Sciences, Curtin School of Population Health, Curtin University, Perth, WA 6102, Australia; 6 Global Centre for Preventive Health and Nutrition (GLOBE), Institute of Health Transformation, School of Health and Social Development, Deakin University, Geelong, VIC 3220, Australia; 7 School of Land and Food Sciences, University of Queensland, Brisbane, QLD 4072, Australia

**Keywords:** innovation, food, aged care, healthcare, sustainability

## Abstract

Population ageing and climate change are issues of global concern. Subsequently, the need for healthy and sustainable food systems to meet the increasing demands for health and aged care is evident. This review aimed to systematically identify studies reporting new or innovative foods, drinks and snack products in health and aged care, and describe health and environmental sustainability considerations where reported. Methods were guided by the Joanna Briggs Institute guidelines for scoping reviews and reported against the PRISMA-ScR guidelines. Eligible studies were conducted in an inpatient healthcare setting or aged care facility where a new or innovative food, drink or snack product was evaluated with outcomes of product use, acceptability, cost, appropriateness for the population, and clinical or environmental sustainability outcomes in the last decade. Three databases were searched using a replicable strategy, with five publications of four studies included in the final library. Product innovations were led at the facility level and included testing dewaxed brown rice, talbinah, and an apple/pear juice fibre solution. Results suggest that food industry suppliers are operating in parallel with foodservices within hospital and aged care. Future intersection would be transformative for both industry sectors.

## 1. Introduction

Each year, an estimated 265 million meals are served to hospitalised patients (n = 190,000 per day) [1] and people living in residential aged care settings (n = 537,000 per day) [2] in Australia. Global food production and delivery in these settings is magnified at scale. According to the Australian and New Zealand Standard Industrial Classification (ANZSIC), the food and beverage sector is in the top three industries that provide agricultural product inputs into the aged care sector, accounting for 18% of all intermediate inputs [3]. The health sector food system that supports the production, procurement, transportation and foodservice to ensure that each meal is delivered is layered with complexity. In addition to reliance on a supply chain which has been shown to be increasingly unstable throughout the COVID pandemic [4], there is an extended array of health conditions to be considered, many of which require therapeutic diets. In hospitals these will vary across the spectrum of disease states, whilst in aged care, dietary needs are focused on reducing nutritional decline. Health and aged care have been identified as sectors where the food system is in particular need of transformation in order to be healthier and more sustainable [5,6].

In this respect, the information and material flow related to food supply is an important component of the health and aged care sector. It entails a complex supply chain made up of multiple stakeholders and operations. Policy commitments to support transforming these components to promote healthy and sustainable food systems in hospital and aged care have been made at state [7] and global [8] levels of governance, however innovation to the food supply and manufacture within this sector has not previously been described. In line with global imperatives to improve food systems to protect human and planetary health [9], the integration of healthy and sustainable food system into health services and aged care is becoming increasingly apparent. Policy statements released by authoritative bodies [9,10] highlight the challenges of influencing planetary health, the health sector and aged care sector, and the globalised food system. However, there is very little information and research on the foods and processing methods required to meet these demands.

An important step in facilitating this food system transition is to examine the interface between the food sector and the aged care sector, identify research needed to meet the challenges faced at the food sector-health/aged care sector intersection, and explore issues such as how to improve the collaboration and coordination, and what partnerships could be fostered and strengthened to facilitate change. Previous authors have already established that innovation is a means to developing healthy and sustainable foods [11]. Accessing appropriate food products is an identified challenge for health service food retailers [12], highlighting the importance of understanding the current and potential food supply of healthy and sustainable food products that are safe and flavoursome. 

To address this research gap, this review aimed to systematically (1) identify studies reporting new or innovative foods, drinks and snack products in health and aged care, and (2) describe health and environmental sustainability considerations where these were reported.

## 2. Materials and Methods

A scoping review was undertaken guided by the Joanna Briggs Institute Manual for Evidence Synthesis (Chapter 11 Scoping Reviews) [13,14] and reported against the Preferred Reporting Items for Systematic reviews and Meta-Analyses extension for Scoping Reviews checklist (PRISMA-ScR) [15]. This design was selected due to the broad scope of the research question, whilst still providing a replicable and transparent process. The review protocol was not prospectively registered.

The inclusion criteria are outlined in Table 1. Studies were eligible if they were conducted in an inpatient healthcare setting including hospitals or an aged care facility. The concept of interest was the trial or evaluation of a new or innovative food, drink or snack product with outcomes of product use, acceptability, cost, appropriateness for the population, and any relevant clinical or environmental sustainability outcomes. To identify relevant new and or innovative products, research publication dates were restricted to the last 10 years (from 1 January 2012). Exclusion criteria included literature that reported a change in the foodservice menu design, food size, composition, or texture changes, fortification of current items with no whole foods added, the product of interest being a pill, tablet, nutraceutical or medication, and the provision of additional oral nutrition support supplements to patients. Peer reviewed papers in any language and study design were eligible. Papers were ineligible if they were letters to the editor, conference abstracts, theses or grey literature.

The following databases were searched on 5 August 2022 via EBSCOHost using a search strategy designed in conjunction with a subject librarian: MEDLINE Complete, Global Health and CINAHL Complete (Table S1: Database search strategies). The search terms utilised three fields relating to hospital and aged care settings, new and innovative products, and food. All keyword search terms were repeated across the three databases and subject headings were customised to suit each individual database selections in line with the themes of the search fields, remaining as similar as possible. All results were downloaded into EndNote (version X9, Clarivate Analytics, Philadelphia) [16] and duplicates were removed before being uploaded to Covidence where additional duplicates were removed by the software (Veritas Health Innovation, Melbourne) [17]. Title and abstract screening was completed independently and in duplicate for 10% of the identified papers with 98% agreement, as such the remainder of the title and abstract screening was completed by one reviewer. The full text screening was completed independently and in duplicate by the same two reviewers where consensus and agreement were reached on any discrepancies that arose. Additionally, the reference lists of the included papers were hand searched for possible eligible papers.

A customised table designed for this review was used to extract data from the included papers. Data were extracted on the following study characteristics: author, year, setting, foodservice type, location, study aim, study design and method, the new or innovative nutrition product used (e.g., food, drink, snack) and its preparation, how this product was implemented in the foodservice delivery, the end user, and any acceptability, clinical or sustainability outcomes reported during the introduction of the product to the foodservice. Data were synthesised narratively. Guided by the JBI Guidance for scoping reviews [13,14] and the PRISMA-ScR guidelines, [15] a quality assessment of included studies was not completed.

## 3. Results

**Figure 1:**
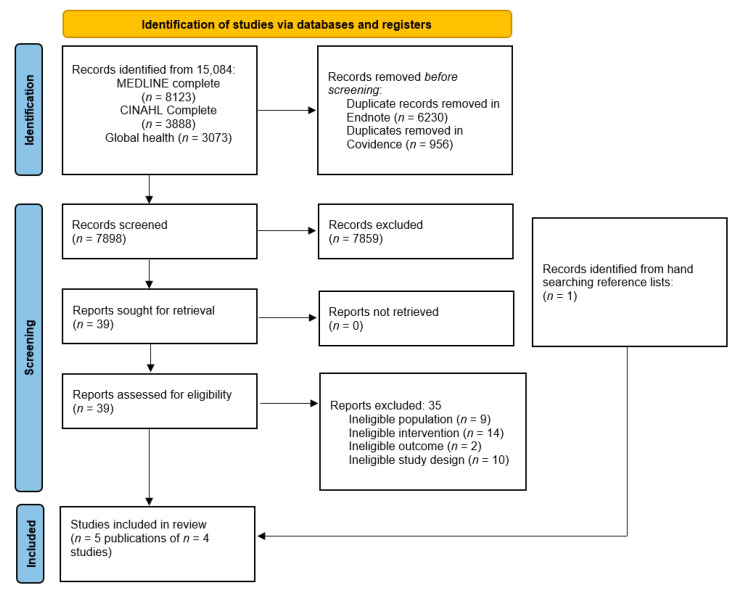
PRISMA flow diagram of included studies for the systematic review of new or innovative nutrition products used in hospital and aged care foodservices.

From the database searches a total of 15,084 articles were located. After removal of duplicates 7898 articles were imported into Covidence [17] for title and abstract screening, documented in Figure 1. Four articles were identified as eligible for inclusion [18,19,20,21]. From the hand searching of included paper reference lists a related article from a previously identified study was eligible [22]. A list of excluded studies and reasons for exclusion are provided in Table S2: Excluded full text references.

Included studies were conducted in Malaysia [18], Japan [20], the United States [21], and Denmark [19,22]. There were two reports contributing to one study, whereby one tested food in the nursing home setting [22] and the other evaluated the sustainability of the items [19]. Three studies were completed in homes for the aged [18,20,22], an academic orthopaedic specialty hospital [21] and to meet the Danish Ministry of Food institution-diet recommendations for elderly populations with poor appetite and dysphagia in nursing homes [19]. The four studies [18,20,21,22] which implemented food product innovations delivered them to depressed older adults, 60 years or older (GDS-R >3) [18], adults 65 years or older [20], dysphagic older adults, over 70 years [22] and postoperative spine fusion patients with an expected length of stay at least three days [21]. Research designs included two randomised trials [18,21], two non-randomised interventional studies [20,22] and one cross sectional study [19].

All studies prepared their new or innovative food, drink or snack products on site at the aged care facility [18,20,22] or hospital [21] with no food industry involvement. A detailed description of the food, preparation method and reasons for implementation are presented in Table 2. The continued use of the innovative or new food were not reported in any study. The foods, drinks and snacks were trialled and then either recommended [18,19,20,22], not recommended [21], or to be further studied for potential use in the future.

Different innovative foods were described by three studies including: dewaxed brown rice (DBR) to improve cognitive function [20], talbinah to decrease depression [18], and an apple and pear juice drink to promote bowel movements [21] (Table 2). Two of the studies [19,22] were based on 20 snack recipes developed to increase the energy and protein intake in elderly Denmark nursing home residents through product innovations such as apple porridge with vanilla cream, tuna mousse and prune trifle.

Clinical outcomes were reported in three of the studies, measuring their outcomes of interest using previously developed patient questionnaires before [18,20], during [18,20] or after [18,20,21] the delivery of their interventions (Table 3).

The dewaxed brown rice study [20] used the Revised Hasegawa’s Dementia Scale to measure age associated dementia. Participants were allocated into a low cognitive or high cognitive function group. There were no significant differences between the consumption of dewaxed brown rice and the control (white rice) at the end of the intervention, however, in the low cognitive function group there were significant increases in their HDS-R scores when the consumption of dewaxed brown rice was compared to white rice. Additionally, compared to their baseline HDS-R scores the low cognitive function group had more improved scores, and less decreased or no changes in scores, when comparing their ingestion of white rice to dewaxed brown rice.

To measure depression, the talbinah (porridge made from barley flour) study [18] used the Geriatric Depression Scale; the total mood disturbances scale; and the depression anxiety and stress scale 21-item questionnaire including the subcategories of depression, stress and anxiety. Mood improvements were identified across the majority of measures.

The apple and pear juice fibre solution study [21] measured participants’ bowel function index and constipation assessment scale scores, time to first bowel motion post-surgery and number of bowel movements between surgery and up to day three post-surgery. There were no significant differences between any of the outcomes measured in this study.

The sustainability profiles of the new or innovative foods, drinks or snacks were reported by one study [19]. The sustainability of the 20 snack foods were measured using a consequential life cycle assessment technique (that identifies the environmental consequences of a decision or a proposed change in the system under study) to describe their environmental impact moving from soil-to-table using 16 impact categories (Table 3) and three functional units, the weight of the foods and their energy (kJ) and protein content. The sustainability of items was reported in total global warming impact measured in kgCO2-eq and the overall monetised environmental impact (EUR) generated through the 16 impact categories where the three most important categories (respiratory inorganics, nature occupation, global warming) were reported separately compared to the sum of the remaining 13 categories. For snacks, both global warming impact and monetised environmental impact were reported. The ten snacks which had lower kgCO2-eq and monetary value (EUR) on average had a 40% less sustainability impact compared to those ranked 11–20 regardless of functional unit chosen. However, when the functional unit changed (energy, protein, weight) the order of the 10 lowest kgCO2-eq producing and monetised environmental impact snacks from the total 20 included reformed to represent different snacks.

Acceptability of the new or innovative food was only reported in one study [22]. Of the 20 food products tasted by residents, vanilla ice cream, strawberry parfait and panna cotta were the most preferred between meals. Foods with added garnishes or layered foods were not significantly preferred over foods without these accompaniments.

## 4. Discussion

This scoping review found that despite the demand for healthy and sustainable food products in health and aged care, the research into the next phase of food innovation is constrained. Whilst these settings are responsible for the dietary intake of a large proportion of the nutritionally dependent population (including individuals with special nutritional requirements), the priority food system components of food procurement and foodservice [23,24] need transformation to protect future population and planetary health [25].

Results suggest that suppliers (food industries including horticulture and food manufacturers) are operating in parallel with the demand (foodservices within hospital and aged care) component of this vast industry. Greater intersection of these industries would be transformative for food in health and aged care. The development of products would have benefits in terms of commercial outcomes, while contributing practical solutions to tackling dietary risk factors and environmental disruptions to food systems. New and emerging technologies for food sector transformation can help to bridge the ‘gap’ between nutritional food standards and the food sectors. These areas include food preservation through new ways to retain nutritional value, safety and flavour; reduced and/or sustainable food packaging; shelf-life improvement, processing of raw food and retaining nutritional value; easy and healthy accessibility (e.g., ready to eat food); and reduction in cosmetic imperfections visible in some farm produce [26,27,28,29].

We suggest that the food industry can optimise its capabilities to create more value-added products that meet consumer and market demand within the context of rapidly evolving interest in health, safety and sustainability considerations [30]. Authors have also identified scope for new food production methods to emerge [31]. Through the types of food and beverages they provide, including to health and aged care facilities, food industries have a critical role to play in implementing evidence-informed healthy and sustainable food supply chains. Despite this need, previous efforts to bring about change have achieved mixed outcomes and have not always been well coordinated with policy guidelines and demands from the health and aged care sectors [32]. One of the reasons for this setback has been the lack of proper consideration for consumer acceptability and factors that influence this acceptability, such as cultural background.

The inclusion of only one study in this review considering the health and sustainability of foods and food systems within health and aged care was surprising [19] as this has been a focus internationally [33,34,35] and locally [36,37] within Australia. There are significant market opportunities for the food industry to meet projected increasing demands for healthy and sustainable food products and practices. Over the next three decades, all regions of the world are expected to experience an unprecedented and sustained change in the age structure of their populations, with the proportion of the global population aged ≥65 years increasing from 9.3% in 2020 to 16.0% in 2050 [38] with associated increases in spending and investment to support the care needs that arise from this ageing population [39]. It has been argued that the general ageing of the population results in an increase in the size of the ‘mature aged’ consumer segment who continue to demand convenient, healthy, and functional food. Hence, there are opportunities to meet the demands of the ageing, health-conscious population through an expanded range of convenient, nutritious, and functional food. Moreover, through the institutional health care sector (including in aged care facilities), there is demand for tasty, nutritious food, including partially prepared foods [40].

Brokering of greater connections between industry, government regulators and health and aged care providers is needed to actively translate this type of nutrition evidence into the development of innovative fortified food products and beverages, that are acceptable to the target market. The availability and consumption of these types of healthier food by the aged care residents will directly and indirectly improve their health and well-being, in turn potentially reducing the costs associated with the provision of medical and related services.

This review has highlighted some opportunities for future research in this sector. Implementation research may support the translation of new products into these settings, particularly through understanding the barriers to implementation. Opportunities also exist for greater engagement with consumers (patients and residents) to understand their food preferences, ensuring alignment with dietary requirements. Multiple research gaps also exist from a sustainability perspective, particularly evaluating the true environmental cost of product development and implementation, which may differ from the economic cost.

This scoping review utilised a wide-ranging search strategy across multiple databases, and was performed and reported against relevant reporting guidelines. Authors acknowledge that the entire library was not reviewed in duplicate and that there is a risk that some research may have been omitted.

## 5. Conclusions

This review found that currently there are few healthy and sustainable food product innovations to support foodservices in health and aged care settings. Into the future it is predicted that demographic shifts will result in an increasing proportion of populations around the world demanding healthy food products in health and aged care settings. It is also anticipated that ongoing environmental disruptions will place increasing demands for the development of these healthy food products to be resilient to changing environmental circumstances and have minimal environmental footprint. Transformations of the food systems within the health and aged care sectors are needed.

## Figures and Tables

**Table 1:** Inclusion criteria.

Criteria	Description
Participants	Health care inpatients and aged care residents
Concept	New or innovative nutrition product for patient consumption including food, drink or snack being trialled/tested, implemented or evaluated; Product acceptability, cost, appropriateness for the population, any relevant clinical or sustainability outcomes
Context	Health service: including hospital (public or private), medical centre, aged care facility (nursing home, retirement home, assisted living facility) that provides food for patients/residents through a foodservice model
Study design	Primary peer reviewed research with any observational or experimental study design including studies using quantitative, qualitative or mixed method data collection, pilot or evaluation study

**Table 2:** Study characteristics of innovative food and drinks implemented in health and aged care.

Author, Year	Population, Setting, Foodservice Type, Location	Study Aim	Study Design; Method	New or Innovative Food, Drink, Snack; Preparation
Baradari et al. 2013 [18]	*n* = 30 depressed (GDS-R > 3) older adults (>60 yrs old), long term care facility, not stated, Malaysia	Measure the effect of talbinah on depression, stress, anxiety and mood	Crossover randomised clinical trial; two groups, one serving of talbinah/day at morning tea vs. control (habitual diet) for 7 wks–3 wk intervention, 1 wk washout, 3 wk intervention where depression (GDS-R, DASS-D), stress (DASS-S), anxiety (DASS-A) and mood (POMS, TMD) were measured at 0, 3, 4 and 7 wks	Talbinah is a traditional Arab food which is ground roasted barley with milk and honey; 25 g ready-made market bought talbinah mixed in 100 mL warm water
Saxe et al. 2017 [19]	Older adults with poor appetite and dysphagia, nursing homes, not stated, Denmark	Measure the environmental impact and sustainability of 20 common snacks developed to meet the Danish institution-diet recommendations for elderly populations with dysphagia and poor appetite and identify which ingredients are responsible for higher environmental impacts	Descriptive cross sectional; Consequential life cycle assessment (cLCA) of each snack recipe including ingredients, cooking, baking, cooling and freezing. The cLCA measured 16 environmental impact categories: the three most important impact categories are presented separately in this study (respiratory inorganics, nature occupation, global warming) and the rest were presented as a sum (human carcinogenic and non-carcinogenic toxicity, ionizing radiation, ozone layer depletion, aquatic and terrestrial ecotoxicity, acidification, aquatic and terrestrial eutrophication, respiratory organics, photochemical ozone effects on vegetation, non-renewable energy and mineral extraction). Snacks were split into two groups, the 10 best and 10 worst as defined by their global warming and monetised environmental impact.	Snacks were: apple porridge with vanilla cream, cauliflower soup, chocolate mousse, lemon mousse, mashed roots with butter, milkshake, protein drink, panna cotta, prune trifle, raspberry jelly with vanilla cream, rice porridge with cinnamon sugar, rum mousse, ryaa high-energy vanilla ice cream, rye bread soup and whipped cream, soup of asparagus with chicken, strawberry parfait, strawberry porridge and vanilla cream, tuna mousse, vegetable soup, yoghurt/strawberry drink. Although some of these foods were fortified using traditional approaches (e.g., cream) many were fortified with fruits, and other core foods to increase their appeal. All snacks have at least 6 g of protein, and 400 kj, and all weighed 100 g; preparation not stated.
Uenobe et al. 2019 [20]	*n* = 31 elderly (>65 yrs old), nursing home, not stated, Japan	Measure the effect of continuous ingestion of DBR on cognitive function improvement	Controlled crossover trial; two groups (low or high cognitive function) ate DBR or WR 3 x/day for approximately 6 mths and then the other rice type for the same period, HDSR-R scores measured at 0, 6 & 12 mths.	DBR is brown rice with only the outermost wax bran layer removed, DBR has 0.81 ng vs. WR 0.04 ng lipopolysaccharides; the WR and DBR were prepared in rice cookers as rice meal or rice porridge. DBR rice meal was prepared with 2–3 parts water added to one-part DBR (g) and rice porridge was cooked using five parts water added to one-part DBR.
Wittig-Wells et al. 2019 [21]	*n* = 46 postoperative orthopaedic patients between 36–82 yrs old, orthopaedic specialty hospital, not stated, United States	Measure the effect of a prune juice and apple juice fibre solution to prevent constipation	Post-test control group randomised control trial; two groups, control and one where patients consumed the dietary fibre solution orally twice a day at 9 am and 9 pm for 3 days post operation, participant constipation was measured using the BFI and CAS on the third night, the total number of bowel movements in the 3 days post operation and time (hrs) to first bowel movement post operation were recorded. Stool softeners and laxatives could be requested by patients.	These juices were chosen due to their recommendation for the prevention and treatment of constipation. The fibre solution comprised 118 mL prune juice and 118 mL apple juice and was heated for 10 s in a microwave; consumption followed 237 mL room temperature water.
Okkels et al. 2018 [22]	*n* = 30 dysphagic elderly (>70 yrs old) from 3 nursing homes; Denmark	Identify the most liked between meal items based on flavour, describe the sensory properties of these items and identify the equality of flavour and appearance-based preferences	Non-randomised interventional study. 20 most popular between meals were chosen from a combination of two hospital and one municipal kitchen menus. 15 min interviews partnered with a 3-point Likert scale questionnaire (bad, neither bad or good, good) asked participants about appetite, appearance and flavour of the items they tasted. Participants tasted 5 items per day across 4 days. Each tasting was separated by a spoon of water.	Between meals were: pumpkin soup, carrot soup, clear soup, mashed potato with bacon and onion, rice porridge with cinnamon sugar, soup of asparagus with chicken, milkshake, rum mousse, apple porridge with vanilla cream, protein drink, rye bread soup with whipped cream, raspberry jelly with vanilla cream, prune trifle, yoghurt/strawberry drink, strawberry porridge with vanilla cream, chocolate mousse, lemon mousse, panna cotta, strawberry parfait, vanilla ice cream. Each item contained between 1.9–7.9 g protein and 165–1409 kJ per portion and all weighed 100 g; these foods were texture modified according to Danish standards (minced and moist, pureed) as required and some recipes were fortified using a protein powder, garnished with sprinkles (e.g., blueberry dust) or layered with other additional foods (e.g., whipped cream). All foods were frozen and thawed the day before service, then served as necessary (e.g., potato was warmed, ice cream was frozen).

**Table 3:** Outcomes of innovative food and drinks implemented in health and aged care.

Author, Year	Clinical Outcomes Reported	Sustainability Outcomes Reported	Acceptability Outcome Reported
Badrasawi et al. 2013 [18]	In the intervention group there was a significant decrease in GDS-R, DASS-D, DASS-S and TMD mean scores post intervention vs. the time without talbinah (*p* = <0.05) All POMS subcategory mean scores except POMS V significantly decreased (*p* = <0.05) No significant changes in any measure for the control group (*p* = >0.05) Significant increase (*p* = <0.05) in calories, zinc and magnesium intake in the intervention group	NR	NR
Saxe et al. 2017 [19]	NR	For individual recipes the global warming impact and monetised overall environmental impact were significantly different between the 10 best and worst snacks On average the best 10 snacks have a 40% less global warming impact and monetised environmental impact than the 10 worst, however snacks change between these two categories depending on weight, energy or protein content. Monetised environmental impacts were contributed by 46% global warming, 26% nature occupation, 12% respiratory inorganics and the remaining sum of the 13 impacts were 16%. Ingredients with the most environmental impacts were cream, and protein powders (protein drinks, whey and casein). Products were packaged on 8 g plastic trays and in 0.5 g plastic film adding a global warming impact of 0.02 kg CO_2_-eq and a monetised environmental impact of 0.002 EUR per meal. The delivery of items via medium vans also added 0.026–0.435 kg CO_2_-eq and 0.005–0.09 EUR per meal.	NR
Uenobe et al. 2019 [20]	No significant difference in total HDS-R score between start and end of either WR or DBR consumption. Significant increase in HDS-R total score for the low cognitive function group consuming DBR compared to WR (*p* = 0.01). Low cognitive function participants consuming DBR had significantly improved HDS-R scores compared to decreased or no change in score from baseline (*p* = 0.17).	NR	NR
Wittig-Wells et al. 2019 [21]	There were no significant differences (*p* = <0.05) between groups BFI or CAS scores. 68.2% of patients had a bowel movement in the first 3 days (intervention group) vs. control 58.3%, not significantly different. Bowel movements ranged from 1 to 9 with the medians for both groups being 1, not significantly different. Time to first bowel movement ranged 17.8–98.7 hrs, intervention group were 59.9 hrs compared to control 70.2 hrs, not significantly different.	NR	NR
Okkels et al. 2018 [22]	NR	NR	Vanilla ice cream, strawberry parfait and panna cotta were the most preferred between meals. Appetite was not significantly associated with flavour ratings. In-between meals with higher fat and energy content but not protein were significantly correlated with higher flavour likings. Lower temperature foods had higher liking and there were significant differences between liking of foods with sprinkles or which were layered. Sour and sweet tasting in-between meals scored significantly higher than salty items. Rum mousse and clear soup had significantly higher appearance scores when compared with their flavour.

GDS-R, Geriatric depression scale–residential, POMS, Profile of mood states, TMD, total mood disturbances, DASS, depression anxiety and stress scale, POMS V, profile of mood stat-vigour, NR, Not reported, DBR, Dewaxed brown rice, WR, White rice, HDS-R, Revised Hasegawa’s dementia scale (HDS-R) score, BFI, bowel function index, CAS, constipation assessment scale.

## Data Availability

Data sharing not applicable. No new data were created or analysed in this study. Data sharing is not applicable to this article.

## Supplementary Materials

The following supporting information can be downloaded at: https://www.mdpi.com/article/10.3390/foods11223604/s1, Table S1: Database search strategies; Table S2: Excluded full text references.

## References

[1] Australia Institute of Health and Welfare (2022). Australia’s Hospitals at a Glance. https://www.aihw.gov.au/reports/hospitals/australias-hospitals-at-a-glance/contents/access-to-hospitals.

[2] Australia Institute of Health and Welfare (2022). People Using Aged Care. https://www.gen-agedcaredata.gov.au/Topics/People-using-aged-care.

[3] Deloitte Access Economics (2016). Australia’s Aged Care Sector: Economic Contribution and Future Directions. https://www2.deloitte.com/content/dam/Deloitte/au/Documents/Economics/deloitte-au-australias-aged-care-sector-economic-contribution-010616.pdf.

[4] Barman A., Das R., De P.K. (2021). Impact of Covid-19 in Food Supply Chain: Disruptions and Recovery Strategy. Curr. Res. Behav. Sci..

[5] Dietitians Association of Australia (2020). Food Systems and Environmental Sustainability Role Statement.

[6] Harvie J., Mikkelsen L., Shak L. (2009). A New Health Care Prevention Agenda: Sustainable Food Procurement and Agricultural Policy. JHEN.

[7] Queensland Health, State of Queensland Government (2017). An Evidence-Based Demand Management Toolkit for Dietetic Services: Framework for Effective and Efficient Dietetic Services (Feeds).

[8] World Health Organization (2021). Action Framework for Developing and Implementing Public Food Procurement and Service Policies for a Healthy Diet.

[9] Watts N., Amann M., Arnell N., Ayeb-Karlsson S., Beagley J., Belesova K., Boykoff P.M., Byass P., Cai W., Campbell-Lendrum D. (2021). The 2020 Report of the Lancet Countdown on Health and Climate Change: Responding to Converging Crises. Lancet.

[10] Barbour L., Bicknell E., Brimblecombe J., Carino S., Fairweather M., Lawrence M., Slattery J., Woods J., World E. (2022). Dietitians Australia Position Statement on Healthy and Sustainable Diets. Nutr. Diet..

[11] Rabadán A., Nieto R., Bernabéu R. (2021). Food Innovation as a Means of Developing Healthier and More Sustainable Foods. Foods.

[12] Law K.K., Pulker C.E., Healy J.D., Pollard C.M. (2021). “Just So You Know, It Has Been Hard”: Food Retailers’ Perspectives of Implementing a Food and Nutrition Policy in Public Healthcare Settings. Nutrients.

[13] Peters M.D., Marnie C., Tricco A.C., Pollock D., Munn Z., Alexander L., McInerney P., Godfrey C.M., Khalil H. (2020). Updated Methodological Guidance for the Conduct of Scoping Reviews. JBI Evid. Synth..

[14] Aromataris E., Munn Z. Jbi Manual for Evidence Synthesis. Jbi, 2020. https://synthesismanual.jbi.global.

[15] Tricco A.C., Lillie E., Zarin W., O’Brien K.K., Colquhoun H., Levac D., Moher D., Peters M.D.J., Horsley T., Weeks L. (2018). Prisma Extension for Scoping Reviews (Prisma-Scr): Checklist and Explanation. Ann. Intern. Med..

[16] The EndNote Team Endnote.

[17] Veritas Health Innovation. Covidence Systematic Review Software, Covidence 2.0 ed..

[18] Badrasawi M.M., Shahar S., Abd Manaf Z., Haron H. (2013). Effect of Talbinah Food Consumption on Depressive Symptoms among Elderly Individuals in Long Term Care Facilities, Randomized Clinical Trial. Clin. Interv. Aging.

[19] Saxe H., Loftager Okkels S., Jensen J.D. (2017). How to Obtain Forty Percent Less Environmental Impact by Healthy, Protein-Optimized Snacks for Older Adults. Int. J. Environ. Res. Public Health.

[20] Uenobe M., Saika T., Waku N., Ohno M., Inagawa H. (2019). Efficacy of Continuous Ingestion of Dewaxed Brown Rice on the Cognitive Functions of the Residents of Elderly Welfare Facilities: A Pilot Test Using Crossover Trial. Food Sci. Nutr..

[21] Wittig-Wells D., Sapp P., Higgins M., Davis E., Carter J., Jacob A. (2019). Randomized Controlled Trial of a Natural Food-Based Fiber Solution to Prevent Constipation in Postoperative Spine Fusion Patients. Orthop. Nurs..

[22] Okkels S., Saxosen M., Bügel S., Olsen A., Klausen T., Beck A. (2018). Acceptance of Texture-Modified in-between-Meals among Old Adults with Dysphagia. Clin. Nutr. ESPEN.

[23] Lawrence M.A., Friel S., Wingrove K., James S.W., Candy S. (2015). Formulating Policy Activities to Promote Healthy and Sustainable Diets. Public Health Nutr..

[24] United Nations System Standing Committee on Nutrition (2016). The Un Decade of Action on Nutrition 2016–2025 Introduction. Rome and Geneva: United Nations Decade of Action on Nutrition Secretariat.

[25] Lawrence M., Friel S. (2019). Healthy and Sustainable Food Systems.

[26] Apeel Sciences Longer Lasting Produce, Plant Base Protection That Helps the Produce You Love Stay Fresh Longer 2022. https://www.apeel.com/about.

[27] Hazel Technologies Inc. Reduce Your Waste, Increase Your Sales 2022. https://www.hazeltechnologies.com/.

[28] Minor T., Gregory A., Sharon Raszap S., Suzanne T., Jean B., Claudia H., Kantor K., Kuchler F., Ellison B., Mishra A. Economic Drivers of Food Loss at the Farm and Pre-Retail Sectors: A Look at the Produce Supply Chain in the United States, Eib-216, 2020. https://www.ers.usda.gov/webdocs/publications/95779/eib-216.pdf.

[29] United Nations Environment Programme The Role of Business Transforming Food Systems UNEP, Nairobi 2021. https://wedocs.unep.org/bitstream/handle/20.500.11822/36755/GEO4B3.pdf.

[30] Food Innovation Australia Limited Capturing the Prize. https://www.fial.com.au/sharing-knowledge/capturing-the-prize.

[31] Ridoutt B. (2023). New Plant-Based and Alternative Protein Foods–Realising the Benefits and Avoiding the Risks. Nutr. Diet..

[32] Alberdi G., Begiristain-Zubillaga M. (2021). Identifying a Sustainable Food Procurement Strategy in Healthcare Systems: A Scoping Review. Sustainability.

[33] Cook N., Goodwin D., Porter J., Collins J. (2022). Food and Food-Related Waste Management Strategies in Hospital Food Services: A Systematic Review. Nutr. Diet..

[34] Carino S., Porter J., Malekpour S., Collins J. (2020). Environmental Sustainability of Hospital Foodservices across the Food Supply Chain: A Systematic Review. J. Acad. Nutr. Diet..

[35] Romanello M., Di Napoli C., Drummond P., Green C., Kennard H., Lampard P., Scamman D., Arnell N., Ayeb-Karlsson S., Ford L.B. (2022). The 2022 Report of the Lancet Countdown on Health and Climate Change: Health at the Mercy of Fossil Fuels. Lancet.

[36] Porter J., Collins J. (2021). A Qualitative Study Exploring Hospital Food Waste from the Patient Perspective. J. Nutr. Educ. Behav..

[37] Neaves B., Bell J.J., McCray S. (2022). Impact of Room Service on Nutritional Intake, Plate and Production Waste, Meal Quality and Patient Satisfaction and Meal Costs: A Single Site Pre-Post Evaluation. Nutr. Diet..

[38] United Nations Department of Economic and Social Affairs, Population Division (2020). World Population Ageing 2020 Highlights: Living Arrangements of Older Persons.

[39] Productivity Commission (2011). Caring for Older Australians: Overview; Report No. 53, Final Inquiry Report.

[40] Spencer S., Kneebone M. Foodmap: An Analysis of the Australian Food Supply Chain, Department of Agriculture, Fisheries and Forestry Canberra 2012. https://www.agriculture.gov.au/sites/default/files/sitecollectiondocuments/ag-food/food/national-food-plan/submissions-received/foodmap-an-analysis-of-the-australian-food-supply-chain-30-july.pdf.

