# Supplementary material for: Innovation in Healthy and Sustainable Food Product Development for Health and Aged Care: A Scoping Review
Source: Foods. 2022 Nov 11;11(22):3604. doi: 10.3390/foods11223604 (PMC9689587; doi:10.3390/foods11223604)
Supplement: Supplementary file 1 [file foods-11-03604-s001.zip › Supplementary file S1 - Table S1 Database search strategies.pdf]

Table S1: Database search strategies

a) **EBSCOHost Medline Complete database search terms for systematic review of new or innovative nutrition products used in hospital and aged care foodservices**

Database	EBSCOHost Medline Complete	
Time frame	2012-Current (5.8.2022)	
Field	Search terms	
1 Hospital or aged care home	Key words	TI hospital or AB hospital or TI hospitals or AB hospitals or TI healthcare* or AB healthcare* or TI "health care*" or AB "health care*" or TI "health service*" or AB "health service*" or TI "medical centre*" or AB "medical centre*" or TI "medical center*" or AB "medical center*" or TI "medical facility*" or AB "medical facility*" or TI "medical facilities" or AB "medical facilities" or TI "home for the aged" or AB "home for the aged" or TI "nursing home" or AB "nursing home" or TI "aged care" or AB "aged care" or TI ((elderly or geriatric or aged or long-term or extended) N3 (care or home* or resident* or institutionali* or "nursing home")) or AB ((elderly or geriatric or aged or long-term or extended) N3 (care or home* or resident* or institutionali* or "nursing home"))
	OR	
	MeSH Headings	(MH "Hospitals") or (MH "Residential Facilities") or (MH "Homes for the Aged") or (MH "Nursing Homes") or (MH "Food Service, Hospital") or (MH "Health Services") or (MH "Long-Term Care") or (MH "Aged") or (MH "Health Care Sector") or (MH "Delivery of Health Care")

<table>
<tr><td colspan="3">AND</td></tr>
<tr><td rowspan="3">2 New or innovative</td><td>Key words</td><td>TI new or AB new or TI innovat* or AB innovat* or TI functional or AB functional or TI novel or AB novel</td></tr>
<tr><td colspan="2">OR</td></tr>
<tr><td>MeSH Headings</td><td>(MH "Creativity") or (MH "Patents as Topic") or (MH "Functional Food")</td></tr>
<tr><td colspan="3">AND</td></tr>
<tr><td rowspan="3">3 Food</td><td>Key Words</td><td>TI food* or AB food* or TI drink* or AB drink* or TI snack* or AB snack*</td></tr>
<tr><td colspan="2">OR</td></tr>
<tr><td>MeSH Headings</td><td>(MH "Food, Fortified") or (MH "Dietary Supplements") or (MH "Food") or (MH "Beverages") or (MH "Food and Beverages") or (MH "Nutrition Therapy") or (MH "Snacks") (MH "Food Industry") or (MH "Food Technology") or (MH "Industry")</td></tr>
</table>

b) EBSCOHost CINAHL Complete database search terms for systematic review of new or innovative nutrition products used in hospital and aged care foodservices

Database	CINAHL Complete	
Time frame	2000-Current (5.8.2022)	
Field	Search terms	
1 Hospital or aged care home	Key words	TI hospital or AB hospital or TI hospitals or AB hospitals or TI healthcare* or AB healthcare* or TI "health care*" or AB "health care*" or TI "health service*" or AB "health service*" or TI "medical centre*" or AB "medical centre*" or TI "medical center*" or AB "medical center*" or TI "medical facility*" or AB "medical facility*" or TI "medical facilities" or AB "medical facilities" or TI "home for the aged" or AB "home for the aged" or TI "nursing home" or AB "nursing home" or TI "aged care" or AB "aged care" or TI ((elderly or geriatric or aged or long-term or extended) N3 (care or home* or resident* or institutionali* or "nursing home")) or AB ((elderly or geriatric or aged or long-term or extended) N3 (care or home* or resident* or institutionali* or "nursing home"))
	OR	
	CINAHL Subject Headings	(MH "Health Facilities") or (MH "Food Service Department") or (MH "Hospitals") or (MH "Health Services") or (MH "Health Services for Older Persons") or (MH "Nursing Homes") or (MH "Residential Facilities") or (MH "Residential Care") or (MH "Long Term Care") or (MH "Gerontologic Care") or (MH "Geriatric Nutrition") or (MH "Health Services for Older

		Persons") or (MH "Aged") or (MH "Housing for Older Persons") or (MH "Food Services")
AND		
2 New or innovative	Key words	TI new or AB new or TI innovat* or AB innovat* or TI functional or AB functional or TI novel or AB novel
	OR	
	CINAHL Subject Headings	(MH "Patents") or (MH "Product Development") or (MH "Functional Food")
AND		
3 Food	Key Words	TI food* or AB food* or TI drink* or AB drink* or TI snack* or AB snack*
	OR	
	CINAHL Subject Headings	(MH "Food") or (MH "Food Technology") or (MH "Food Industry") or (MH "Dietary Supplements") or (MH "Food and Beverages") or (MH "Snacks") or (MH "Meals") or (MH "Beverages")

c) EBSCOHost Global Health database search terms for systematic review of new or innovative nutrition products used in hospital and aged care foodservices

Database	Global Health	
Time frame	2012-Current (5.8.2022)	
Field	Search terms	
1 Hospital or aged care home	Key words	TI hospital or AB hospital or TI hospitals or AB hospitals or TI healthcare* or AB healthcare* or TI "health care*" or AB "health care*" or TI "health service*" or AB "health service*" or TI "medical centre*" or AB "medical centre*" or TI "medical center*" or AB "medical center*" or TI "medical facility*" or AB "medical facility*" or TI "medical facilities" or AB "medical facilities" or TI "home for the aged" or AB "home for the aged" or TI "nursing home" or AB "nursing home" or TI "aged care" or AB "aged care" or TI ((elderly or geriatric or aged or long-term or extended) N3 (care or home* or resident* or institutionali* or "nursing home")) or AB ((elderly or geriatric or aged or long-term or extended) N3 (care or home* or resident* or institutionali* or "nursing home"))
	OR	
	CAB Subject Thesaurus	(DE "hospitals") or (DE "health services") or (DE "hospital care") or (DE "hospital catering") or (DE "nursing homes") or (DE "long term care") or (DE "institutional catering") or (DE "elderly") or (DE "elderly nutrition")
AND		
	Key words	TI new or AB new or TI innovat* or AB innovat* or TI functional or AB functional or TI novel or AB novel

2 New or innovative	OR	
	CAB Subject Thesaurus	(DE "new products") or (DE "innovations") (DE "patents") or (DE "product development") or (DE "production possibilities") or (DE "novel foods") or (DE "functional foods")
AND		
3 Food	Key Words	TI food* or AB food* or TI drink* or AB drink* or TI snack* or AB snack*
	OR	
	CAB Subject Thesaurus	(DE "food") or (DE "food products") or (DE "foods") or (DE "beverages") or (DE "snacks") or (DE "meals") or (DE "food supplements") or (DE "food industry")
