# Supplementary material for: Innovation in Healthy and Sustainable Food Product Development for Health and Aged Care: A Scoping Review
Source: Foods. 2022 Nov 11;11(22):3604. doi: 10.3390/foods11223604 (PMC9689587; doi:10.3390/foods11223604)
Supplement: Supplementary file 1 [file foods-11-03604-s001.zip › Supplementary file S2 - Table S2 Excluded full text references.pdf]

Table S2: Excluded full-text references (n = 33)

Reason for ineligibility	References
Ineligible population (e.g. not healthcare or aged care) – n = 9	41. Baugreet, S.; Kerry, J.P., Botineştean, C.; Allen, P.; Hamill, R.M. Development of novel fortified beef patties with added functional protein ingredients for the elderly. *Meat Sci* **2016**, *122*, 40–47.<br>42. Chao, C.; Hwang, J.S.; Kim, I.W.; Choi, R.Y.; Kim, H.W.; Park, H.J. Coaxial 3D printing of chicken surimi incorporated with mealworm protein isolate as texture-modified food for the elderly. *J.Food Eng.* 2022, *333,* Doi:10.1016/j.jfoodeng.2022.111151.<br>43. García, J.; Méndez, D.; Álvarez, M.; Sanmartin, B.; Vázquez, R.; Regueiro, L.; Atanassova, M. Design of novel functional food products enriched with bioactive extracts from holothurians for meeting the nutritional needs of the elderly. *LWT-Food Sci. Tech.* **2019**, *109*, 55–62.<br>44. Kennedy, K.; Keogh, B.; Lopez, C.; Adelfio, A.; Molloy, B.; Kerr, A.; Wall, A.; Jalowicki, G.; Holton, T. Khaldi, N. An artificial intelligence characterised functional ingredient, derived from rice, inhibits TNF-α and significantly improves physical strength in an inflammaging population. *Foods* **2020**, *9*, 1147.<br>45. Magno, S.; Ceccarini, G.; Pelosini, C.; Jaccheri, R.; Vitti, J.; Fierabracci, P.; Salvetti, G.; Airoldi, G.; Minale, M.; Saponati, G.; Santini, F. LDL-cholesterol lowering effect of a new dietary supplement: an open label, controlled, randomized, cross-over clinical trial in patients with mild-to-moderate hypercholesterolemia. *Lipid Health Dis.* **2018**, *17*, 124.<br>46. Millar, C.L.; Duclos, Q.; Garcia, C.; Norris, G.H.; Lemos, B.S.; DiMarco, D.M.; Fernandez, M.L.; Blesso, C.N. Effects of Freeze-Dried Grape Powder on High-Density Lipoprotein Function in Adults with Metabolic Syndrome: A Randomized Controlled Pilot Study. *Metab. Syndr. Relat. Disord.* **2018**, *16*, 464–469.<br>47. Muramatsu, K.; Mitsuboshi, S. Development of a new Itohiki-Natto manufactured for elderly Japanese. *Nippon Shokuhin Kagaku Kogaku Kaishi = J. Jpn. Soc. Food Sci. Tech.* **2013**, *60*, 381–386.<br>48. Nguyen, J.Y.; Rock, C.R.; Gray, V.B.; Claver, M.; Costa, C. Product development considerations of flaxseed supplementation for the aging population: a pilot study. *J. Food. Res.* **2019**, *8*, 42–49.<br>49. Setiawan, B.; Aulia, S.S.; Sinaga, T.; Sulaeman, A. Nutritional content and characteristics of pumpkin cream soup with tempeh addition as supplementary food for elderly. *Int.J. Food Sci.* **2021**, Doi:10.1155/2021/6976357.

Ineligible intervention (e.g. not new or innovative nutrition product) – *n* = 12	50. Beelen, J.; de Roos, N.M.; de Groot, L.C.P.G.M. Protein enrichment of familiar foods as an innovative strategy to increase protein intake in institutionalized elderly. *J. Nutr. Health Aging* **2017**, *21*, 173–179.<br>51. Cicolari, S.; Pavanello, C.; Olmastroni, E.; Del Puppo, M.; Bertolotti , M.; Mombelli, G.; Catapano, A.L.; Calabresi, L.; Magni, P. Interactions of Oxysterols with Atherosclerosis Biomarkers in Subjects with Moderate Hypercholesterolemia and Effects of a Nutraceutical Combination (Bifidobacterium longum BB536, Red Yeast Rice Extract) (Randomized, Double-Blind, Placebo-Controlled Study). *Nutrients* **2021**, *13*, 427.<br>52. Costarelli, L.; Giacconi, R.; Malavolta, M.; Basso, A.; Piacenza, F.; DeMartiis, M.; Giannandrea, E.; Renieri, C.; Busco, F.; Galeazzi, R.; et al. Effects of zinc-fortified drinking skim milk (as functional food) on cytokine release and thymic hormone activity in very old persons: a pilot study. *Age* **2014**, *36*, 1421–1431.<br>53. Iuliano, S.; Poon, S.; Robbins, J.; Bui, M.; Wang, X.; De Groot, L.; Van Loan, M.; Ghasem Zadeh, A.; Nguyen, T.; Seeman, E. Effect of dietary sources of calcium and protein on hip fractures and falls in older adults in residential care: cluster randomised controlled trial. *BMJ* **2021,** *375*, n2364.<br>54. Lassen, A.D.; Nordman, M.; Christensen, L.M.; Beck, A.M.; Trolle, E. Guidance for Healthy and More Climate-Friendly Diets in Nursing Homes—Scenario Analysis Based on a Municipality's Food Procurement. *Nutrients* **2021**, *13*, 4525–4525.<br>55. Myint, M.W.W.; Wu, J.; Wong, E.; Chan, S.P.; To, T.S.J.; Chau, M.W.R.; Ting, K.H.; Fung, P.M.; Au, K.S.D. Clinical benefits of oral nutritional supplementation for elderly hip fracture patients: a single blind randomised controlled trial. *Age Ageing* **2013**, *42*, 39–45.<br>56. Maki, K.C.; Geohas, J.G.; Dicklin, M.R.; Huebner, M.; Udani, J.K. Safety and lipid-altering efficacy of a new omega-3 fatty acid and antioxidant-containing medical food in men and women with elevated triacylglycerols. *Prostaglandins Leukotrienes Essent. Fatty Acid* **2015**, *99*, 41–46.<br>57. Neelemaat, F.; Bosmans, J.E.; Thijs, A.; Seidell, J.C.; van Bokhorst-de van der Schuerena, M.A.E. Oral nutritional support in malnourished elderly decreases functional limitations with no extra costs. *Clin. Nutrition (Edinburgh, Scotland)* **2012**, *31*, 183–190.<br>58. Pilipenko, V.I.; Teplyuk, D.A.; Shakhovskaya, A.K.; Isakov, V.A.; Vorobyova, V.M.; Vorobyova, I.S.; Sarkisyan, V.A.; Kochetkova, A.A.; Mikheeva, G.A.; Yudina, A.V. Using a multicomponent functional food in IBS patients with constipation a comparative controlled study. *Voprosy Pitaniia* **2016**, *85*, 84–91.

	59. Rambousková, J.; Procházka, B.; Binder, M.; Anděl, M. Effect of the liquid milk nutritional supplement with enhanced content of whey protein on the nutritional status of the elderly. *Vnitrni Lekarstvi* **2014**, *60*, 556–561.<br>60. Rasmussen, J. The LipiDiDiet trial: what does it add to the current evidence for Fortasyn Connect in early Alzheimer's disease? *Clin. Interv. Aging* **2019**, *14*, 1481–1492.<br>61. Van Wymelbeke, V.; Brondel, L.; Bon, F.; Martin-Pfitzenmeyer, I.; Manckoundia, P. An innovative brioche enriched in protein and energy improves the nutritional status of malnourished nursing home residents compared to oral nutritional supplement and usual breakfast: FARINE+ project. *Clin. Nutr. ESPEN* **2016**, *15*, 93–100.
Ineligible outcome (e.g. not acceptability, cost, appropriateness) – n = 2	62. Leong, P.; Basham, J.E.; Yong, T.; Chazan, A.; Finlay, P.; Barnes, S.; Bardin, P.G.; Campbell, D. A double blind randomized placebo control crossover trial on the effect of dietary nitrate supplementation on exercise tolerance in stable moderate chronic obstructive pulmonary disease. *BMC Pulm. Med.* **2015**, *15*, 52.<br>63. De Oliveira, L.P.L.; de Seixas Filho, J.T.; Pereira, M.M.; Mello, S.C.R.P. Frog meat in special diets: potential for use as a functional food. *Bol Inst. Pesca* **2017**, *43*, 99–106.
Ineligible outcome (e.g. review, conference abstract) – n = 10	64. Beelen, J.; de Roos, N.; de Groot, L. SUN-LB025: Protein Enrichment of Familiar Foods as an Innovative Strategy to Increase Protein Intake in Institutionalized Elderly. *Clin. Nutr.* **2015**, *34*, S244–S244.<br>65. Durakova, A.; Gogova, T.; Vladeva, S.; Vasileva, A.; Slavov, A.; Yanakieva, V.; Temelkova, M. Biscuits with flour of lucuma, spelt and carob for prophylactic and dietary nutrition. *Journal of Hygienic Eng. Des.* **2021**, *37*, 174–179.<br>66. Kahathuduwa, C.N.; Allison, D.B. Insufficient Reporting of Randomization Procedures and Unexplained Unequal Allocation: A Commentary on "Dairy-Based and Energy-Enriched Berry-Based Snacks Improve or Maintain Nutritional and Functional Status in Older People in Home Care". *J. Nutr. Health Aging.* **2019**, *23*, 396. doi:10.1007/s12603-019-1183-0.<br>67. Nykanen, I. Letter to the editor: Insufficient Reporting of Randomization Procedures and Unexplained Unequal Allocation: A Commentary on "Dairy-Based and Energy-Enriched Berry-Based Snacks Improve or Maintain Nutritional and Functional Status in Older People in Home Care". *J. Nutr. Health Aging.* **2019**, *23*, 397. doi:10.1007/s12603-019-1184-z.<br>68. Kurt, E.L. Conference Report. New Plant-Derived Drugs from Traditional Chinese Medicine Discussed at UN Conference. Available

	online:https://www.herbalgram.org/resources/herbalgram/issues/107/table-of-contents/hg107-confrpt-ilsc/ (accessed on 1 Spetember 2022).<br>69. Lee, J. Changes on the menu. Increasing emphasis on wellness and healthier dietary choices leads to new directions in hospital food service. *Mod. Healthc.* **2013**, *43*, 28–29.<br>70. Oboh, G. Phytomedicine and functional foods: Keys to sustainable healthcare delivery. *J. Food Biochem.* **2021**, *45*, e13634.<br>71. Sarvananda, L.; Premarathna, A.D.; Shafras, M. Applications of micro- and macroalgae elements in the food and healthcare industries. *CAB Rev.* **2021**, *16*, 1–10.<br>72. Shahidi, F. Special Issue: Omega-3 lipids. *J. Funct. Food* **2015**, *19*, 797–982.<br>73. Uenobe, M.; Saika, T.; Waku, N. OHNO, M.; INAGAWA, H. Effect of Continuous Dewaxed Brown Rice Ingestion on the Cognitive Function of Elderly Individuals. *J. Nutr. Sci Vitam.* **2019**, *65*, S122–S124.
